# LXRα improves myocardial glucose tolerance and reduces cardiac hypertrophy in a mouse model of obesity-induced type 2 diabetes

**DOI:** 10.1007/s00125-015-3827-x

**Published:** 2015-12-18

**Authors:** Megan V. Cannon, Herman H. W. Silljé, Jürgen W. A. Sijbesma, Mohsin A. F. Khan, Knut R. Steffensen, Wiek H. van Gilst, Rudolf A. de Boer

**Affiliations:** University of Groningen, University Medical Center Groningen, Department of Cardiology, Hanzeplein 1, 9713 GZ Groningen, the Netherlands; University of Groningen, University Medical Center Groningen, Department of Nuclear Medicine, Hanzeplein 1, 9713 GZ Groningen, the Netherlands; Division of Clinical Chemistry, Department of Laboratory Medicine, Karolinska Institutet, Karolinska University Hospital, Stockholm, Sweden

**Keywords:** Diabetic cardiomyopathy, Left ventricular hypertrophy, Liver X receptor, Natriuretic peptides

## Abstract

**Aims/hypothesis:**

Diabetic cardiomyopathy is a myocardial disease triggered by impaired insulin signalling, increased fatty acid uptake and diminished glucose utilisation. Liver X receptors (LXRs) are key transcriptional regulators of metabolic homeostasis. However, their effect in the diabetic heart is largely unknown.

**Methods:**

We cloned murine *Lxrα* (also known as *Nr1h3*) behind the α-myosin heavy chain (*αMhc*; also known as *Myh6*) promoter to create transgenic (*Lxrα*-Tg) mice and transgene-negative littermates (wild-type [WT]). A mouse model of type 2 diabetes was induced by a high-fat diet (HFD, 60% energy from fat) over 16 weeks and compared with a low-fat diet (10% energy from fat). A mouse model of type 1 diabetes was induced via streptozotocin injection over 12 weeks.

**Results:**

HFD manifested comparable increases in body weight, plasma triacylglycerol and insulin resistance per OGTT in *Lxrα*-Tg and WT mice. HFD significantly increased left ventricular weight by 21% in WT hearts, but only by 5% in *Lxrα*-Tg. To elucidate metabolic effects in the heart, microPET (positron emission tomography) imaging revealed that cardiac glucose uptake was increased by 1.4-fold in WT mice on an HFD, but further augmented by 1.7-fold in *Lxrα*-Tg hearts, in part through 5′ adenosine monophosphate-activated protein kinase (AMPK) phosphorylation and restoration of glucose transporter 4 (GLUT4). By contrast, streptozotocin-induced ablation of insulin signalling diminished cardiac glucose uptake levels and caused cardiac dysfunction, indicating that insulin may be important in LXRα-mediated glucose uptake. Chromatin immunoprecipitation assays identified natriuretic peptides, atrial natriuretic peptide (ANP) and B-type natriuretic peptide (BNP), as potential direct targets of cardiac LXRα overexpression.

**Conclusions/interpretation:**

Cardiac-specific LXRα overexpression ameliorates the progression of HFD-induced left ventricular hypertrophy in association with increased glucose reliance and natriuretic peptide signalling during the early phase of diabetic cardiomyopathy. These findings implicate a potential protective role for LXR in targeting metabolic disturbances underlying diabetes.

**Electronic supplementary material:**

The online version of this article (doi:10.1007/s00125-015-3827-x) contains peer-reviewed but unedited supplementary material, which is available to authorised users.

## Introduction

Metabolic abnormalities such as insulin resistance, disturbed glucose homeostasis, dyslipidaemia and obesity collectively predispose individuals toward the development of type 2 diabetes and are associated with an increased risk of cardiovascular disease and heart failure [1]. Moreover, obesity promotes left ventricular (LV) hypertrophy independent of hypertension [2], and LV hypertrophy is not uncommon in normotensive individuals with diabetes [3]. The underlying pathogenesis of myocardial disease induced by diabetes, referred to as diabetic cardiomyopathy, is only partially understood. However, aberrant myocardial metabolism is implicated in the early manifestation of the disease, as increased circulating fatty acids (FA) and impaired insulin signalling cause a shift in substrate usage towards exclusively favouring FA over glucose. The consequent lack of metabolic flexibility leads to lipotoxicity, impaired calcium signalling and mitochondrial dysfunction, which manifests as increased myocardial stiffness, hypertrophy and diastolic dysfunction [4].

Liver X receptors α and β (LXRα and LXRβ) are sterol-activated transcription factors belonging to the nuclear receptor superfamily. LXRs activate target gene transcription through heterodimerization with the retinoid X receptor (RXR) and by interacting with an LXR response element (LXRE). LXRs have emerged as central regulators of cholesterol homeostasis and lipid and glucose metabolism, and have established anti-inflammatory and immune functions. In insulin-resistant diabetic rodents, synthetic LXR activation has been shown to reduce hyperglycaemia [5, 6] and improve peripheral insulin sensitivity [7, 8], effects that are mediated across multi-organ systems including suppression of gluconeogenic genes in the liver and improved peripheral glucose disposal in adipose tissue [5, 7] and skeletal muscle [9]. LXRs also play an important role in the normal and diabetic kidney through regulation of intracellular cholesterol and inflammation [10]. In the heart, activation of LXRs has been shown to attenuate pathological cardiac hypertrophy [11–13], ischaemia/reperfusion injury [14, 15], and very recently, diabetic cardiomyopathy in a *db/db* mouse model of type 2 diabetes [16].

We have previously shown that mice with selective overexpression of LXRα in the heart (*Lxrα*-Tg) demonstrate increased capacity for myocardial glucose uptake that protects against cardiac dysfunction and adverse remodelling in the adaptation to hypertrophic stress [17]. To date, the metabolic effects of LXRs in the diabetic heart have not been described. Here, we investigate the metabolic and functional consequences of cardiac LXRα activation in response to a metabolic challenge imposed by high-fat diet (HFD)-induced obesity and insulin resistance.

## Methods

A detailed description of methods is provided in the electronic supplementary material (ESM Methods).

### Generation of *Lxrα*-Tg mice

Transgenic mice with cardiac-specific LXRα overexpression were created by cloning a full-length murine *Lxrα* (also known as *Nr1h3*) complementary DNA (cDNA) construct downstream of the cardiac-specific α-myosin heavy chain (*αMhc*; also known as *Myh6*) promoter, as previously described [17]. Mice were bred on a C57BL/6 background and backcrossed for six generations. Nontransgenic littermates (wild-type, WT) served as controls.

### Experimental protocol

Animal studies were performed in accordance with the principles of laboratory animal care (NIH publication no. 85-23, revised 1985) and with approval by the Institutional Animal Care and Use Committee of the University of Groningen, Groningen, the Netherlands. To induce a model of type 2 diabetes, diet intervention commenced in male mice of approximately 12 weeks of age; they received either an HFD (60% energy from fat) or a nutrient-equivalent low-fat control diet (LFD; 10% energy from fat) for 16 weeks. To induce a model of type 1 diabetes over a 12-week period, a low-dose streptozotocin (STZ) induction protocol was performed (50 mg/kg STZ administered intraperitoneally for 5 consecutive days).

Cardiac function was assessed with echocardiography and invasive haemodynamic monitoring, and myocardial glucose uptake was determined using 2-deoxy-2-[^18^F]fluoro-d-glucose ([^18^F]FDG) and microPET (positron emission tomography) imaging, as previously described [17, 18]. An OGTT was performed whereby mice were challenged with a glucose bolus (2 g/kg) and blood glucose levels were measured across a 3 h time course. LV tissue samples were used to perform quantitative real-time PCR and immunoblotting, biochemical assays and histological analysis, as described previously [17].

### Chromatin immunoprecipitation assay

Chromatin immunoprecipitation (ChIP) experiments were performed in both isolated neonatal rat ventricular myocytes and in hearts from *Lxrα*-Tg and WT mice using the Pierce Agarose ChIP Kit (Thermo Scientific, Rockford, IL, USA).

### Statistics

Data are expressed as means ± SEM. For group comparisons, one-way ANOVA was performed followed by Tukey’s post hoc analysis. When the data were not normally distributed according to Shapiro–Wilk test for normality, Kruskal–Wallis test followed by a Mann–Whitney *U* test for individual comparison of means were performed. A value of *p* < 0.05 was considered statistically significant. Statistical analyses were performed using IBM SPSS Statistics 22 (Chicago, IL, USA).

## Results

### HFD induces obesity and insulin resistance in mice

**Fig. 1:**
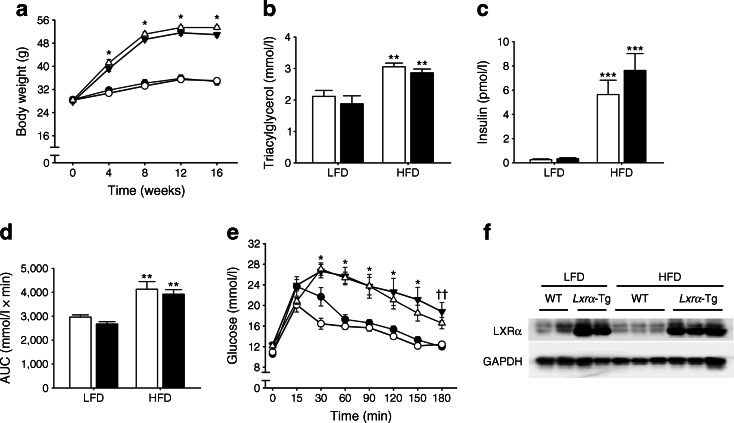
*Lxrα*-Tg and WT mice develop obesity and insulin resistance with high-fat feeding. (**a**, **e**) Circles, LFD; triangles, HFD; black symbols, WT; white symbols, *Lxrα*-Tg . (**b**–**d**) White bars, WT; black bars, *Lxrα*-Tg . (**a**) Body weight increased in mice after 16 weeks of an HFD (*n* = 11–12/group). Measurements of fasted plasma (**b**) triacylglycerol and (**c**) insulin levels (*n* = 8–12/group). (**d**, **e**) OGTTs were performed in mice at 14 weeks (*n* = 9–10/group). (**d**) AUC was calculated from (**e**) serial glucose measurements. All data are means ± SEM. *p < 0.05 vs respective LFD control for both *Lxrα*-Tg and WT; **p < 0.01, ***p < 0.001 vs respective LFD control; ^††^ *p* < 0.01 WT LFD vs WT HFD. (**f**) Western blot of LV LXRα protein expression in mice subjected to LFD or HFD; glyceraldehyde 3-phosphate dehydrogenase (GAPDH) served as loading control

Prior to diet intervention, all *Lxrα*-Tg mice in the LFD and HFD groups displayed comparable measures of body weight with respect to WT mice (Fig. 1a). Obesity developed similarly between *Lxrα*-Tg and WT mice on HFD as both groups gained significant and proportional increases in body weight in the first 8 weeks; by 16 weeks, mice attained approximately 50% of their original body weight (Table 1). Mice receiving an HFD exhibited hypertriglyceridaemia and hyperinsulinaemia, as both circulating triacylglycerol (Fig. 1b) and insulin levels (Fig. 1c) were significantly elevated compared with respective LFD controls. Mice on an HFD were normoglycaemic, yet demonstrated glucose intolerance and insulin resistance per OGTT (Fig. 1d, e). AUC (Fig. 1d) was calculated from response to oral glucose challenge (Fig. 1e). In both HFD groups, post-mortem analysis revealed significant increases in liver weight of 46% and 50% for WT-HFD and *Lxrα*-Tg -HFD, respectively (Table 1). These data suggest that both *Lxrα*-Tg and WT mice incurred comparable systemic effects from HFD intervention, resembling human insulin resistance and impaired glucose tolerance/fasting glucose. The HFD did not affect ventricular LXRα protein expression as assessed by western blot (Fig. 1f).

**Table 1:** Biometric, echocardiographic and haemodynamic variables of *Lxrα*-Tg and WT mice after 16 weeks of an LFD or HFD

Variable	LFD		HFD	
	WT	*Lxrα*-Tg	WT	*Lxrα*-Tg
Post-mortem organ weight	(*n* = 11)	(*n* = 11)	(*n* = 12)	(*n* = 12)
Body weight (g)	34.8 ± 1.3	35.0 ± 0.9	50.9 ± 0.8***	53.4 ± 0.7***
LV/body weight (mg/g)	3.5 ± 0.1	3.2 ± 0.2	2.8 ± 0.1***	2.2 ± 0.2***^,†††^
LV/tibia (mg/mm)	6.9 ± 0.1	6.3 ± 0.2	8.3 ± 0.2***	6.6 ± 0.5*^,†††^
Kidney/tibia (mg/mm)	23.5 ± 0.7	24.6 ± 0.8	25.1 ± 0.6	26.5 ± 0.6
Liver/tibia (mg/mm)	92.7 ± 5.8	89.8 ± 6.4	172.8 ± 9.1***	182.6 ± 7.0***
Echocardiography	(*n* = 11)	(*n* = 10)	(*n* = 12)	(*n* = 12)
Heart rate (bpm)	436 ± 10	436 ± 12	418 ± 9	456 ± 13
LVCO/BW (ml min^−1^ g^−1^)	0.81 ± 0.04	0.72 ± 0.09	0.67 ± 0.04	0.70 ± 0.04
Stroke volume (μl)	66.3 ± 2.6	63.8 ± 2.7	82.9 ± 5.0*	82.3 ± 4.4**
Fractional shortening (%)	40.9 ± 1.5	41.9 ± 1.1	35.2 ± 2.2	36.4 ± 1.3
LV posterior wall, diastole (mm)	0.78 ± 0.03	0.80 ± 0.04	1.10 ± 0.19**	0.88 ± 0.02***
LV posterior wall, systole (mm)	1.43 ± 0.05	1.51 ± 0.09	1.45 ± 0.06	1.40 ± 0.05
Interventricular septum, diastole (mm)	0.78 ± 0.02	0.72 ± 0.01	0.98 ± 0.05**	0.92 ± 0.04***^,†††^
Interventricular septum, systole (mm)	1.48 ± 0.04	1.41 ± 0.05	1.60 ± 0.07	1.56 ± 0.06
LV internal diameter, diastole (mm)	4.00 ± 0.10	3.73 ± 0.11	4.02 ± 0.13	4.26 ± 0.07**
LV internal diameter, systole (mm)	2.37 ± 0.10	2.17 ± 0.09	2.60 ± 0.13	2.63 ± 0.12*
E velocity (m/s)	0.76 ± 0.02	0.71 ± 0.02	0.71 ± 0.03	0.77 ± 0.07
A velocity (m/s)	0.54 ± 0.02	0.51 ± 0.03	0.47 ± 0.03	0.52 ± 0.06
E/A ratio	1.41 ± 0.03	1.43 ± 0.06	1.59 ± 0.11	1.57 ± 0.10
Deceleration time (ms)	44.6 ± 2.8	36.7 ± 2.5	43.8 ± 4.1	37.4 ± 2.4
Haemodynamics	(*n* = 11)	(*n* = 11)	(*n* = 12)	(*n* = 12)
Aortic pressures				
Systolic (mmHg)	101.1 ± 2.2	92.1 ± 2.7	105.8 ± 2.6	105.8 ± 2.3**
Diastolic (mmHg)	66.5 ± 1.9	63.8 ± 1.8	69.9 ± 2.0	70.4 ± 1.4
Mean arterial pressure (mmHg)	78.0 ± 2.0	73.2 ± 2.1	81.9 ± 2.2	82.2 ± 1.6*
Intra-ventricular pressures				
LV end-systolic pressure (mmHg)	99.8 ± 2.0	91.5 ± 2.7	110.7 ± 4.0	103.2 ± 2.6*
LV end-diastolic pressure (mmHg)	7.6 ± 2.2	7.2 ± 1.4	10.4 ± 1.2	13.5 ± 2.0
dP/dt_*max*_ (mmHg)	8,360 ± 363	8,420 ± 305	8,389 ± 375	8,263 ± 293
dP/dt_*min*_ (mmHg)	−7,785 ± 365	−7,433 ± 265	−8,135 ± 348	−7,197 ± 340

Data are expressed as means ± SEM

*p < 0.05, **p < 0.01, ***p < 0.001, HFD versus corresponding LFD group; ^†††^ *p* < 0.001 WT vs *Lxrα*-Tg mice bpm, beats per minute; LVCO, left ventricular cardiac output; BW, body weight

### Cardiac LXRα overexpression prevents development of LV hypertrophy induced by HFD

**Fig. 2:**
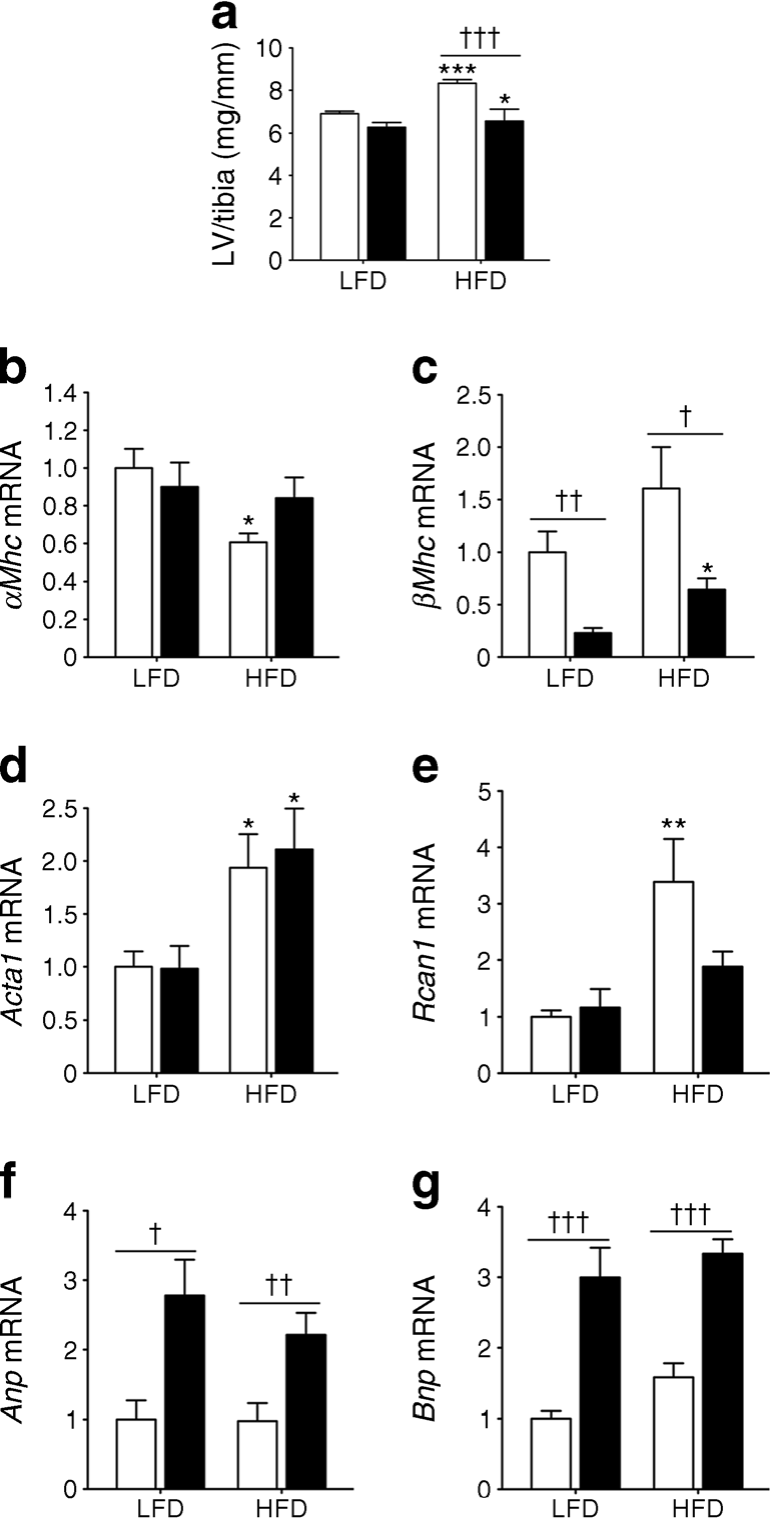
Cardiac-specific LXRα overexpression prevents obesity-induced cardiac hypertrophy. WT (white bars), *Lxrα*-Tg (black bars). (**a**) LV to tibia length ratios in WT and *Lxrα*-Tg mice fed an HFD for 16 weeks; *n* = 11–12/group. (**b**–**g**) Measurement of mRNA levels to assess hypertrophic gene expression. Values are normalised to the invariant transcript, *36b4*, and are expressed as fold change; *n* = 8–10/group. Data are means ± SEM; *p < 0.05, **p < 0.01, ***p < 0.001 vs respective LFD control, ^†^ *p* < 0.05, ^††^ *p* < 0.01, ^†††^ *p* < 0.001 vs WT

HFD feeding over 16 weeks caused a significant increase of 21% in LV weight of WT mice, but only 5% in *Lxrα*-Tg mice (Fig. 2a). Expression of the adult cardiac gene, *αMhc*, was significantly downregulated in WT mice on an HFD (Fig. 2b), whereas the fetal isoform, *βMhc* (also known as *Myh7*), was significantly higher in WT than in *Lxrα*-Tg mice (Fig. 2c). Transcript levels of skeletal muscle alpha-actin (*Acta1*) were increased similarly in both HFD groups (Fig. 2d), whereas regulator of calcineurin 1 (*Rcan1*) was significantly induced in WT but not in *Lxrα*-Tg mice (Fig. 2e). Interestingly, natriuretic peptides, atrial natriuretic peptide (*Anp*, also known as *Nppa*) and B-type natriuretic peptide (*Bnp*, also known as *Nppb*) were upregulated in *Lxrα*-Tg hearts, irrespective of diet (Fig. 2f, g).

Potential growth pathways implicated in cardiac hypertrophy and diabetic cardiomyopathy were also studied. Phosphorylated Akt^Ser473^ protein levels were moderately upregulated similarly between *Lxrα*-Tg and WT mice on HFD (ESM Fig. 1a). In both HFD groups, downstream effects of Akt signalling strongly phosphorylated and activated the ribosomal protein S6 kinase more than threefold, with moderate effects on P70S6 kinase signalling (ESM Fig. 1b, c). HFD intervention did not result in upregulation of pathological extracellular signal-regulated kinase (ERK) and phosphorylated p44/42 mitogen-activated protein kinase (MAPK) pathways (ESM Fig. 1d). These data suggest that phospho-Akt^Ser473^–S6 signalling mediates cardiac growth due to HFD in both *Lxrα*-Tg and WT mice, but is antagonised by the antihypertrophic effects of enhanced natriuretic peptides in hearts overexpressing cardiac LXRα.

### HFD causes mild hypertension and borderline diastolic and systolic dysfunction

Echocardiographic and invasive haemodynamic variables of cardiac function are presented in Table 1. Mean arterial pressure and intracardiac pressures did not differ significantly between *Lxrα*-Tg and WT mice. Blood pressure levels tended to be lower in *Lxrα*-Tg mice and increased with HFD, but not in WT mice. Mice on an HFD did not display signs of diastolic dysfunction, as mitral filling velocities and deceleration time were unaltered, as well as end-diastolic LV pressure and contractility. These data indicate that the associated HFD-induced LV hypertrophy represents early structural remodelling since functional consequences are absent at this time point.

### *Lxrα*-Tg mice demonstrate increased cardiac glucose uptake despite systemic insulin resistance

**Fig. 3:**
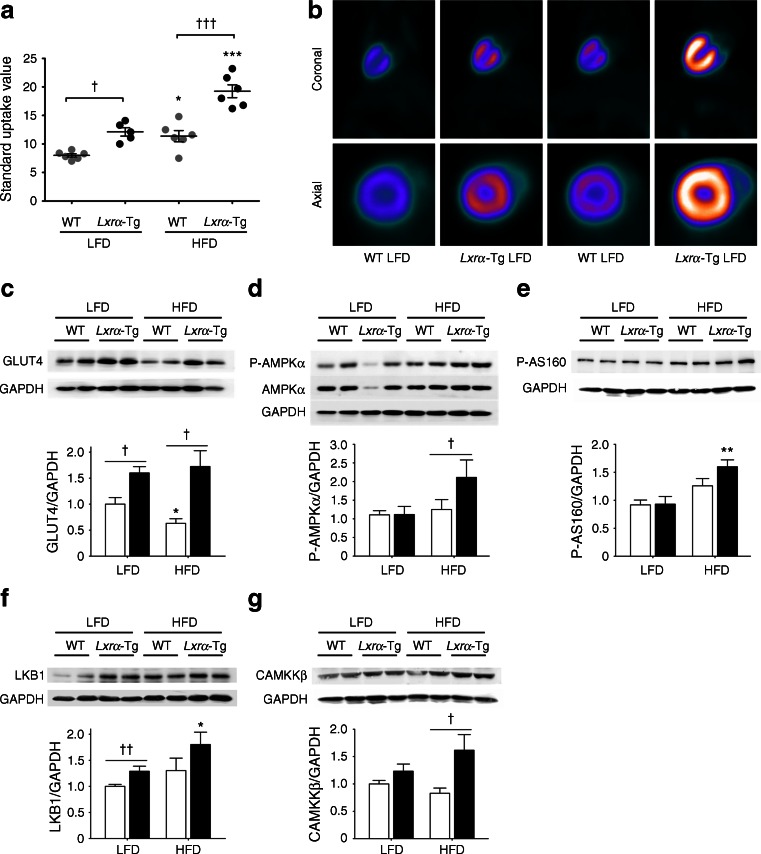
Cardiac LXRα overexpression enhances myocardial glucose uptake in response to an HFD. (**a**, **b**) Mice on either an LFD or HFD underwent [^18^F]FDG and microPET imaging to record myocardial glucose uptake after 16 weeks on respective diets. (**a**) [^18^F]FDG uptake was determined as standard uptake value; *n* = 5–6/group. (**b**) Representative PET images in coronal and axial planes. (**c**–**g**) WT (white bars), *Lxrα*-Tg (black bars). Quantification of (**c**) GLUT4, (**d**) AMPKα phosphorylation, (**e**) AS160 phosphorylation, (**f**) LKB1 and (**g**) CAMKKβ protein levels in LV tissue normalised to GAPDH; *n* = 8/group, except GLUT4 *n* = 5–6/group. Data are means ± SEM; *p < 0.05, **p < 0.01, ***p < 0.001 vs respective LFD control, ^†^ *p* < 0.05, ^††^ *p* < 0.01, ^†††^ *p* < 0.001 vs WT

We have previously demonstrated enhanced myocardial glucose uptake in *Lxrα*-Tg mice and in response to chronic pressure overload-induced hypertrophy [17]. In this study, we tested the functionality of this adaptation by subjecting mice to a metabolic challenge of insulin resistance and hypertriglyceridaemia. Cardiac glucose uptake was significantly increased by 1.5-fold in *Lxrα*-Tg mice on an LFD compared with WT mice. HFD caused a 1.4-fold increase in glucose uptake in WT mice, but this was more markedly enhanced in *Lxrα*-Tg hearts (Fig. 3a, b). LV protein levels of the insulin-dependent glucose transporter 4 (GLUT4) were assessed by western blot (Fig. 3c). GLUT4 was significantly upregulated by 1.6-fold in *Lxrα*-Tg mice on an LFD. However, an HFD significantly suppressed GLUT4 expression in WT mice, but this was restored by LXRα overexpression.

In response to an HFD, phosphorylated 5′ adenosine monophosphate-activated protein kinase (AMPK) was significantly increased by 1.7-fold in *Lxrα*-Tg mice compared with WT (Fig. 3d). Downstream Akt substrate of 160 kDa (AS160) phosphorylation levels were also elevated in diabetic *Lxrα*-Tg hearts, but not in WT-HFD hearts (Fig. 3e). Assessment of upstream kinases of AMPK revealed that liver kinase B1 (LKB1) expression was induced in *Lxrα*-Tg mice both at baseline and on an HFD, but not in WT mice (Fig. 3f), whereas Ca^2+^/calmodulin-dependent protein kinase kinase-beta (CAMKKβ) expression was increased by twofold in *Lxrα*-Tg-HFD hearts compared with WT-HFD hearts (Fig. 3g), implicating a role in Ca^2+^- or contraction-stimulated glucose uptake.

Overall, these data indicate that increased basal cardiac glucose uptake in *Lxrα*-Tg mice is associated with induction of the insulin-dependent GLUT4 transporter, and AMPK phosphorylation may contribute to the enhanced glucose uptake levels following an HFD. Hearts overexpressing cardiac LXRα respond to HFD intervention by upregulating LKB1 and CAMMKβ, which converge to activate AMPK and promote GLUT4 translocation and upregulation via phospho-AS160.

In separate experiments, a model of type 1 diabetes was implemented (ESM Table 1, ESM Fig. 2). To further examine the effects of insulin signalling on LXRα-mediated myocardial glucose uptake, mice were rendered insulin-deficient via STZ treatment. STZ caused a reduction of fasting insulin levels by approximately 50% (ESM Table 1). As a consequence, myocardial glucose uptake levels were severely depressed in both WT and *Lxrα*-Tg mice treated with STZ (ESM Fig. 2), which was associated with a decline in cardiac function (ESM Table 1). In *Lxrα*-Tg hearts subjected to STZ, the inability to upregulate phosphorylated AMPK levels was associated with significant reductions in GLUT4 expression (ESM Fig. 2c, d). Taken together, cardiac LXRα overexpression augments cardiac glucose uptake despite systemic insulin resistance. Nevertheless, the presence of insulin may be necessary for an LXRα-mediated increase in glucose levels.

### Cardiac LXRα overexpression induces transcriptional changes in lipid metabolism

**Fig. 4:**
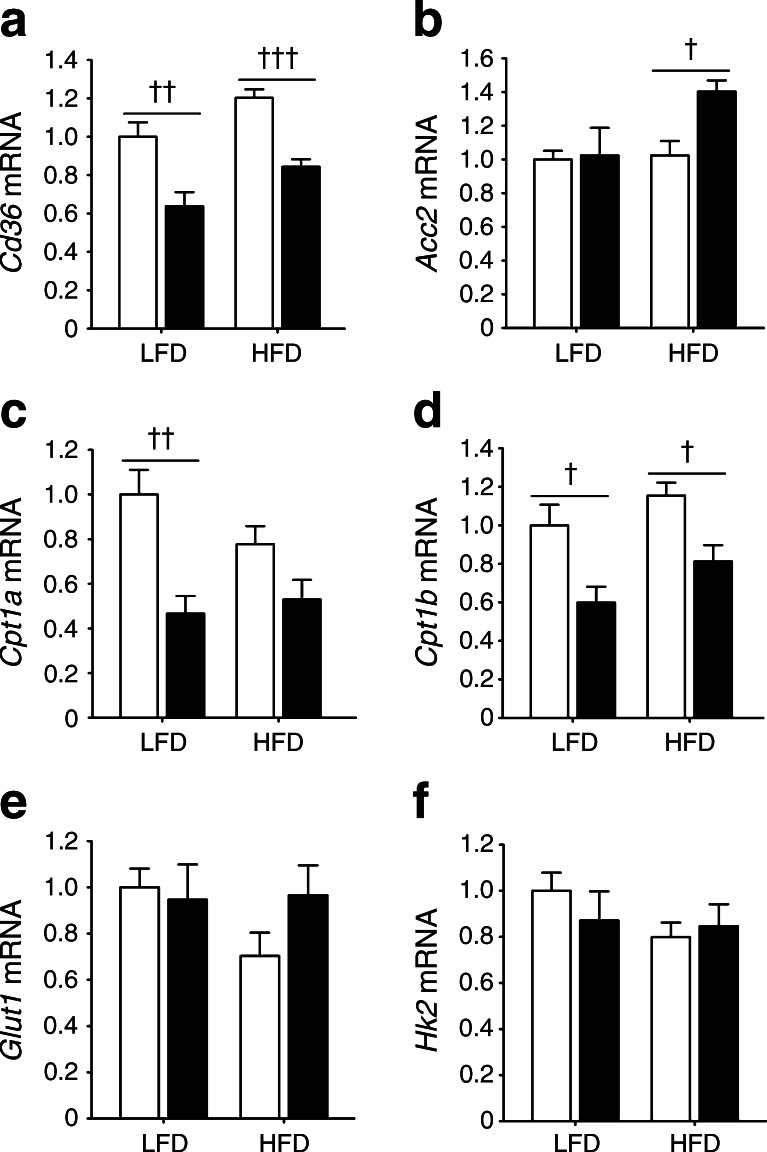
Metabolic gene profile of LXRα transgenic hearts subjected to an HFD. (**a**–**f**) WT (white bars), *Lxrα*-Tg (black bars). mRNA levels were determined in LV samples following 16 weeks of either LFD or HFD for genes involved in (**a**) fatty acid uptake, (**b**–**d**) mitochondrial import and oxidation and (**e**, **f**) glucose uptake and metabolism. mRNA levels are normalised to *36b4*, and expressed as fold change; *n* = 8–10/group. Data are means ± SEM; ^†^ *p* < 0.05, ^††^ *p* < 0.01, ^†††^ *p* < 0.001 vs WT

Genes promoting cellular and mitochondrial FA uptake such as fatty acid translocase (*Cd36*; Fig. 4a) and carnitine parmitoyltransferase I (*Cpt1a* and *Cpt1b*; Fig. 4c, d) were significantly downregulated in *Lxrα*-Tg hearts in response to an HFD, suggesting a deviated shift from FA oxidation pathways. Cardiac LXRα negatively regulates CD36 expression and positively regulates acetyl-CoA carboxylase 2 (ACC2) expression (via an unknown mechanism; Fig. 4b). On the other hand, cardiac LXRα also promotes AMPK signalling (Fig. 3d), which potentially could promote FA oxidation via translocation of CD36 and ACC2 phosphorylation. HFD did not cause substantial changes in cardiac triacylglycerols, although these levels tended to be lower in *Lxrα*-Tg mice (ESM Fig. 3). No detectable differences in the constitutive glucose transporter 1 (*Glut1*; also known as *Slc2a1*) and hexokinase II (*Hk2*) mRNA levels were observed between *Lxrα*-Tg and WT mice (Fig. 4e, f).

### Natriuretic peptides are potential direct targets of LXRα activation

Natriuretic peptides, ANP and BNP, are induced in response to cardiac stress or injury and are antihypertrophic in their effects on the heart [19]. However, their concentrations are decreased in obesity and type 2 diabetes despite the presence of cardiac hypertrophy and dysfunction [20]. LXRs may play a regulatory role in natriuretic peptide expression since *Anp* and *Bnp* mRNA levels are significantly increased by 2.8-fold and 3.0-fold, respectively, in mice with cardiac-specific LXRα overexpression (described previously, [17]), and remain upregulated in the presence of HFD-induced obesity (Fig. 2f, g).

**Fig. 5:**
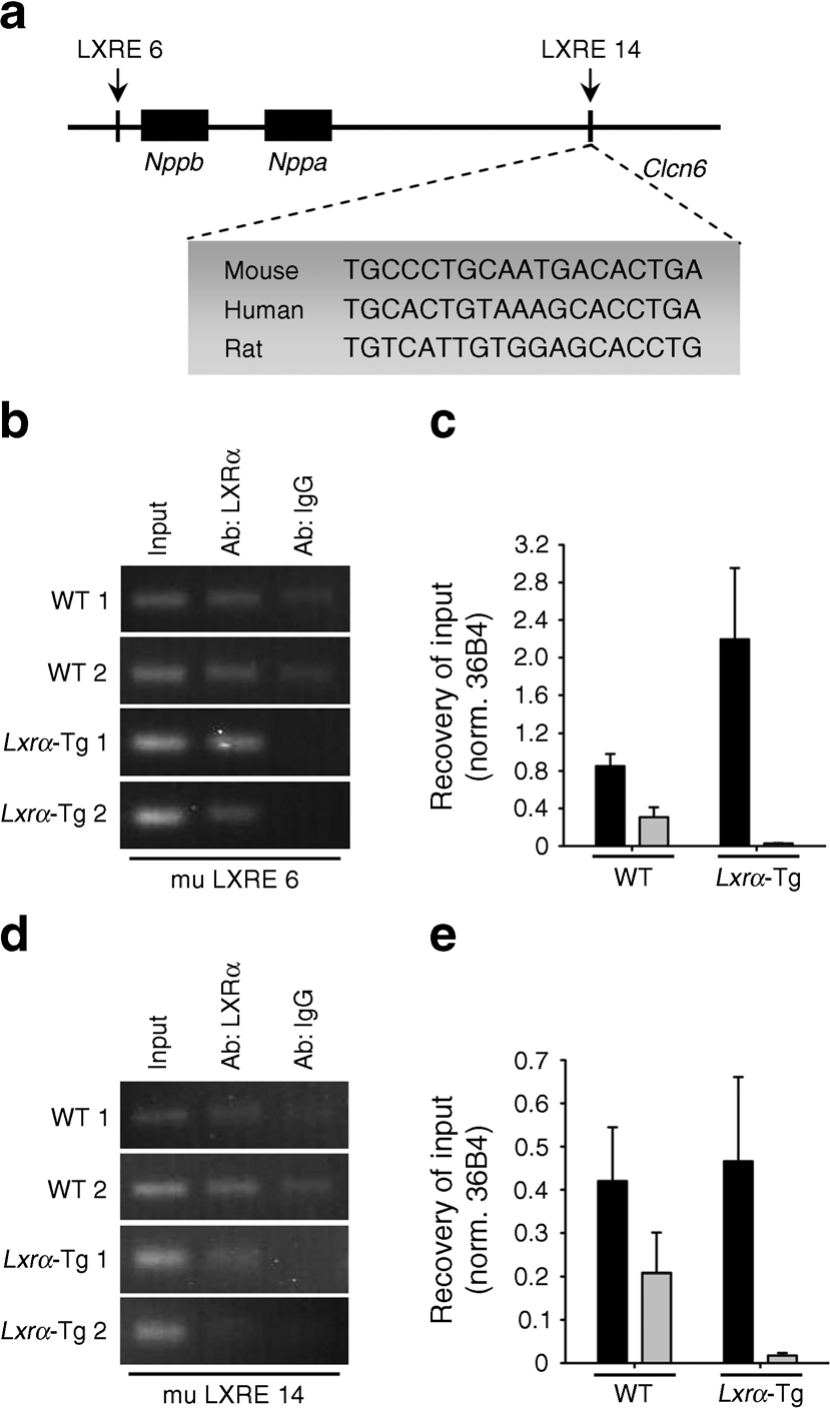
Regulation of natriuretic peptide transcription by LXRα. (**a**) LXRE 14 binding site sequences for mouse, human and rat were identified using the JASPAR database. A region of ±50 kb from the TSS of the *Anp* gene in mice was used together with syntenic regions in humans and rats. Results from this analysis revealed a highly conserved LXRE 14 binding site in the intronic region of *Clcn6* in both mouse and human with a lower degree of conservation in the rat. (**b**–**e**) Chromatin immunoprecipitation assays were performed in heart samples from WT and *Lxrα*-Tg mice to analyse the ANP/BNP region using antibodies directed against LXRα. Analysis of immunoprecipitated chromatin fragments are from two murine preparations per genotype, each preparation representing two pooled hearts, normalised (norm.) to 36B4. (**c**, **e**) Ab:LXRα (black bars), Ab:IgG (grey bars). The results are representative of the real-time PCR fragments and are analysed from gel electrophoresis. Nonspecific IgG antibodies served as a negative control. Ab, antibody; mu, murine

To further evaluate the role of LXRα on myocardial natriuretic peptide transcription, in silico analysis of the ANP/BNP region was performed using algorithms specific for the identification of LXR binding sites, or LXREs. In the mouse, several potential LXREs were identified within ± 50 kb of the *Anp* transcriptional start site (TSS). To determine whether the LXR/RXR heterodimer is recruited to the ANP/BNP region, ChIP analysis was performed in murine heart tissue with antibodies specific for LXRα. An LXRα antibody was sufficient to pull down LXRE-containing DNA fragments of two potential LXREs, LXRE 6 and LXRE 14 (Fig. 5a–e). Agarose gel electrophoresis indicated that recruitment of LXRα to the ANP/BNP region occurred in both WT and *Lxrα*-Tg mice. This gel-based assay was used for quantitative assessment of LXRE fragments by determining abundance of precipitated genomic sequences by real-time PCR. IgG antibodies served as a negative control, confirming lack of an interaction with the LXRE under investigation.

To assess whether LXRE 14 is conserved, putative binding sites for LXRE 14 in mouse, human and rat were identified using the JASPAR database (http://jaspar.genereg.net; Fig. 5a). A region of ± 50 kb from the TSS of the mouse *Anp* gene was used together with syntenic regions in the human and rat. These sequences were then scanned against all available position frequency matrices using a relative profile score threshold of 80%. Results from this analysis revealed an occurrence of the LXRE 14 binding site in the intronic region of *Clcn6*, approximately 30 kb downstream of the TSS of mouse *Anp* (score, 89%). Comparison with orthologous regions in the human and rat identified this site as being highly conserved in human (score, 81%). Relaxing the cut-off of the relative profile score to 70% identified LXRE 14 as being less stringently conserved in rat (score, 74%). Using ChIP assay, we further validated binding of LXRα to this site in isolated rat cardiomyocytes (ESM Fig. 4a, b). LXRE 6 featured as a two-way binding site that was highly conserved in mice (score, 90% and 80%) and rats (score, 87% and 75%), but not in humans (ESM Fig. 4c).

In conclusion, LXRα may serve as an enhancer that binds specifically to the ANP/BNP region to regulate natriuretic peptide expression and mediate its antihypertrophic effects in the heart. Since the *Anp* and *Bnp* genes do not overlap genomically, but rather lie relatively close to each other, it is likely that functional LXREs located within this locus may influence the transcription of either gene or both.

## Discussion

LXRs have been implicated as potential drug targets for the treatment of diabetes and metabolic disorders given their role in improving glucose tolerance and insulin resistance [5, 7, 8, 21]. In the present study, we investigated whether cardiac LXRα protects the heart against diabetic cardiomyopathy. We found that cardiac-specific LXRα overexpression in murine hearts prevented the development of obesity-induced LV hypertrophy in the absence of overt cardiac dysfunction. Despite hyperinsulinaemia and peripheral insulin resistance, myocardial glucose uptake was remarkably enhanced in *Lxrα*-Tg mice on an HFD coincident with increased AMPK activation and restoration of GLUT4.

Diabetic cardiomyopathy has become increasingly recognised as a distinct clinical entity that is characterised by the presence of cardiovascular damage in diabetic patients [3]. Although the existence of several risk factors associated with diabetes, such as hypertension and coronary artery disease, may amplify the effect of diabetes on the heart, diabetes nevertheless incurs adverse changes to the myocardium, including LV hypertrophy and fibrosis, in the absence of these confounding factors [22]. Mice with cardiac-specific LXRα overexpression are protected from LV hypertrophy and dysfunction following hypertrophic perturbations such as chronic pressure overload and angiotensin II stimulation [17]. Here, we extend upon these observations demonstrating that the hypertrophic response is further abrogated following a metabolic challenge of chronic hypertriglyceridaemia and hyperinsulinaemia. HFD intervention induced LV hypertrophy and molecular determinants of hypertrophic stress in WT mice in the absence of fibrosis (data not shown) and cardiac dysfunction, suggesting that hypertrophic remodelling is a very early structural manifestation of cardiomyopathic onset and progression. However, with longer duration of HFD, we speculate that obesity-induced cardiac hypertrophy may predispose mice to cardiac dysfunction, as LV hypertrophy is one of the main precursors of heart failure. Recently, the LXR agonist GW3965 was reported to attenuate fibrosis and apoptosis and improve cardiac function in diabetic *db/db* mice [16]. However, it is important to note that GW3965 also lowered body weight as well as hyperglycaemia and hypercholesterolaemia in these mice whilst improving glucose tolerance and insulin sensitivity [16], suggesting that the beneficial effects of LXR agonism in the heart are likely to be a result of less stress emanating from these systemic metabolic disturbances. In our study, these systemic variables were comparable between WT and *Lxrα*-Tg groups, allowing strictly for heart-specific evaluation of LXR activation.

To date, the metabolic effects of LXR in the diabetic heart have not been described. Metabolic derangements caused by HFD involve increased delivery of FA to the heart, which steers substrate preference towards exclusively FA; this adaptation, coupled with insulin resistance, limits the dependence on glucose. The heart is thus an inadvertent target of diabetes [23]. In this study, we tested the functionality of the cardiac LXRα transgene on myocardial glucose uptake capacity by rendering mice either insulin resistant or deficient. In response to HFD-induced insulin resistance, *Lxrα*-Tg mice nevertheless displayed increased myocardial glucose uptake, mediated in part by restoration of insulin-dependent GLUT4 through increased AMPK phosphorylation. By contrast, diabetes invoked by STZ-induced insulin deficiency led to impaired glucose uptake and cardiac dysfunction, indicating that cardiac LXRα overexpression may not be adequate to improve glucose uptake capacity in the setting of type 1 diabetes.

Interestingly, the HFD tended to mildly increase cardiac glucose uptake, which is consistent with a previous report showing that HFD-induced hyperinsulinaemia augmented glucose uptake in mice in the absence of cardiac dysfunction, and moreover, this increase in glucose flux was critical for preserving mitochondrial function [24]. Preceding the predominant utilisation of lipids that is a hallmark of diabetic cardiomyopathy, the early phase of metabolic remodelling may indeed be characterised by a heightened sensitivity to insulin that promotes glucose uptake and usage. It remains to be determined when the myocardium gradually develops insulin resistance in the face of systemic insulin resistance. Thus far, data from clinical studies are conflicting as myocardial glucose supply is reported to be either unchanged [25] or reduced [26] in diabetic patients.

Maintaining myocardial sensitivity to glucose, or reducing FA uptake may nevertheless be key in preventing the progression of diabetes-associated pathogenesis. Impaired *Glut4* transcription is linked to states of insulin resistance [27]. In light of this, increasing GLUT4 levels may be cardioprotective. In this study, phosphorylation and activation of AMPK by upstream kinases, LKB1 and CAMKKβ, promoted GLUT4 upregulation and translocation via AS160 phosphorylation in *Lxrα*-Tg hearts subjected to an HFD, but not in WT. Other studies support direct effects of LXR agonism on *Glut4* transcription in adipose tissue and skeletal muscle, which enhance peripheral glucose clearance in rodent models of diabetes [5, 7, 9]. Moreover, expression of the FA uptake transporter gene, *Cd36*, is reciprocally downregulated in *Lxrα*-Tg hearts, which may confer antihypertrophic effects since *Cd36* knockout mice are resistant to HFD-induced cardiac hypertrophy [28]. By contrast, increased CD36 expression in middle-aged mice contributes to cardiac hypertrophy, dysfunction and myocardial lipid accumulation [29].

Besides modulation of myocardial substrate metabolism, targeting of natriuretic peptides beyond their established effects on pressure–volume homeostasis may be important in preventing diabetic cardiomyopathy. A cardiometabolic link has been postulated for natriuretic peptides [20], and BNP is implicated in improved glucose utilisation through increased capillary permeability [30]. Natriuretic peptides are also known to antagonise cardiac hypertrophy independently of blood pressure [31, 32], and in humans, certain ANP- and BNP-receptor polymorphisms have been associated with LV mass in essential hypertension [33]. Both *Anp* and *Bnp* are significantly upregulated with cardiac LXRα overexpression and their induction is unaffected by HFD-induced obesity. By contrast, natriuretic peptides are deficient in obesity and diabetes, as obese and insulin-resistant individuals display reduced circulating ANP and BNP levels [34, 35], despite the fact that these metabolic disorders increase the risk of developing cardiovascular disease and heart failure. HFD intervention did not affect natriuretic peptide expression; however, this may be model-dependant as other preclinical studies show that ANP and BNP are downregulated in diabetic *db/db* mice [36, 37]. It is currently unknown why natriuretic peptides are dysregulated in this setting. However, treatment with BNP has been shown to prevent cardiac dysfunction in *db/db* mice by inhibiting cardiac hypertrophy, fibrosis and apoptosis [37], as well as the acute hypertrophic response in the diabetic rat heart [38]. LXRα may therefore prevent obesity-induced cardiac hypertrophy via increased local natriuretic peptide signalling. Unravelling the mechanisms by which LXRα modulates *Anp* and *Bnp* transcription may indeed be complex: activation of the hexosamine biosynthetic pathway through increased glucose flux leads to downstream *O*-linked glycosylation of transcriptional activators of ANP and BNP [17]. Additionally, this study identified putative LXREs in regulatory regions of the ANP/BNP promoter and ChIP analysis confirmed recruitment of LXRα to this region, implicating natriuretic peptides as direct, heart-specific gene targets of LXRα.

Since the structural consequences of diabetes-imposed metabolic stress on the heart are slow and progressive effects, and the clinical manifestation of symptoms is gradual, an understanding of the initial derangements is essential for early intervention. Herein, we capture a very early stage in the pathogenesis of diabetic cardiomyopathy and show that cardiac hypertrophy and glucose response to metabolic stress are preliminary developments. Promoting glucose uptake as well as natriuretic peptide signalling in the heart may be important initiatives in counteracting the progression of diabetes-induced myocardial disease. Interestingly, the recently published PARADIGM study [39] demonstrated the beneficial effects of LCZ696 in patients with heart failure. LCZ696 increases circulating BNP levels and it would be of particular interest to test this novel drug in patients with diabetes. Altogether, our results support the notion that targeting LXRα may be advantageous for intervening in aberrant metabolic signalling, which is a hallmark of cardiovascular disease.

## Electronic supplementary material

ESM Methods (PDF 477 kb)

ESM Fig. 1 (PDF 367 kb)

ESM Fig. 2 (PDF 349 kb)

ESM Fig. 3 (PDF 378 kb)

ESM Fig. 4 (PDF 225 kb)

ESM Table 1 (PDF 455 kb)

## Abbreviations

ACC2: Acetyl-CoA carboxylase 2
AMPK: 5′ Adenosine monophosphate-activated protein kinase
ANP: Atrial natriuretic peptide
AS160: Akt substrate of 160 kDa
BNP: B-type natriuretic peptide
CAMKKβ: Ca^2+^/calmodulin-dependent protein kinase kinase-beta
CD36: Fatty acid translocase
ChIP: Chromatin immunoprecipitation
FA: Fatty acid
[^18^F]FDG: 2-deoxy-2-[^18^F]fluoro-d-glucose
GLUT4: Glucose transporter 4
HFD: High-fat diet
LFD: Low-fat diet
LKB1: Liver kinase B1
LV: Left ventricular
LXR: Liver X receptor
LXRE: Liver X receptor response element
PET: Positron emission tomography
RXR: Retinoid X receptor
STZ: Streptozotocin
TSS: Transcriptional start site
WT: Wild-type
