# Supplementary material for: LXRα improves myocardial glucose tolerance and reduces cardiac hypertrophy in a mouse model of obesity-induced type 2 diabetes
Source: Diabetologia. 2015 Dec 18;59:634–43. doi: 10.1007/s00125-015-3827-x (PMC4742491; doi:10.1007/s00125-015-3827-x)
Supplement: Supplementary file 1 — (PDF 477 kb) [file 125_2015_3827_MOESM1_ESM.pdf]

Supplemental Methods

Generation of *Lxrα*-Tg mice

Transgenic mice with cardiac-specific LXRα overexpression were created by cloning a full-length murine *Lxrα* (also known as *Nr1h3*) complementary DNA (cDNA) construct downstream of the cardiac-specific α-myosin heavy chain (*αMhc*) promoter, as previously described [1]. Mice were bred on a C57BL/6 background and backcrossed for six generations. Non-transgenic littermates (wild-type, WT) served as controls.

Experimental protocol

Animal studies were performed in accordance with the principles of laboratory animal care (NIH publication no. 85-23, revised 1985; http://grants1.nih.gov/grants/olaw/references/phspol.htm) and with approval by the Institutional Animal Care and Use Committee of the University of Groningen, Groningen, the Netherlands. Male mice were housed in a pathogen free, temperature-controlled environment, and maintained on a 12hr:12hr light:dark cycle with *ad libitum* access to water and chow. To induce a model of type 2 diabetes, diet intervention commenced in mice of approximately 12 weeks of age: they received either a high-fat diet (HFD; 60% of energy from fat), or a nutrient-equivalent low-fat control diet (LFD; 10% of energy from fat) for 16 weeks (D12492 and D12450B, respectively, Research Diets Inc., New Jersey, USA).

To induce a model of type 1 diabetes, a low-dose streptozotocin (STZ) induction protocol was performed. Briefly, mice were fasted for 4 hours, followed by an intraperitoneal injection of 50 mg/kg STZ dissolved in sodium citrate buffer that was performed for 5 consecutive days. Control mice received sodium citrate intraperitoneally. After 4 weeks, mice were tested for presence of sufficient hyperglycemia, and blood glucose levels were monitored every 4 weeks thereafter. STZ was initiated over a period of 12 weeks during which time mice were maintained on normal chow (Harlan, The Netherlands).

Oral glucose tolerance test

Mice were fasted for 6 hours followed by administration of a glucose bolus (2 g/kg) by oral gavage. Blood was sampled from the tail vein immediately before the glucose challenge, as well as at 15, 30, 60, 90, 120, 150, and 180 minutes thereafter. Blood glucose levels were determined using Accu-Chek Aviva glucose analyzer (Roche Diagnostics, Mannheim, Germany). The AUC for each time point was calculated using GraphPad Prism (Version 5.04).

Cardiac functional assessment

Transthoracic echocardiography was performed in anesthetized mice using the GE Vivid 7 equipped with 14-MHz transducer (GE Healthcare, Chalfont St. Giles, UK), as previously described [2]. M-mode was recorded in parasternal short axis view to measure septal and posterior wall thickness as well as left ventricular (LV) internal dimensions in diastole and systole, from which percent fractional shortening was determined. In apical views, pulsed wave Doppler was used to measure mitral filling velocities. For determination of LV cardiac output (LVCO), the diameter of the LV outflow tract (LVOT) was measured in parasternal long axis, and pulsed wave Doppler was used to record flow gradients in the LVOT in apical 5-chamber view.

Invasive hemodynamic recordings were obtained with a microtip pressure catheter (1.0 F; Millar Instruments, Houston, TX, USA), described previously [2]. Under anesthesia, the right carotid artery was located and punctured, followed by advancement of the catheter into the proximal aorta for arterial pressure recordings, then into the LV for measurement of intracardiac pressures. From these recordings, heart rate, aortic pressures, LV end-systolic and end-diastolic pressures, and maximal and minimal first derivatives of force (dP/dt_{max} and dP/dt_{min}) were determined. Preceding hemodynamic measurements, mice were fasted for 4 hours. Following hemodynamic monitoring, blood was sampled via heart puncture. The hearts were flushed with phosphate-buffered saline, harvested, and weighed. The LV was portioned

for immunohistochemistry preparations, or frozen in liquid nitrogen and stored at -80°C for biochemical and expressional analyses.

Small animal positron emission tomography (PET) studies

MicroPET imaging of 2-deoxy-2-[^{18}F]fluoro-D-glucose ([^{18}F]-FDG) was used to assess myocardial glucose uptake and was performed on a Focus 220 microPET system (Siemens, USA), as previously described [1]. In brief, food was removed from cages 3 hours prior to imaging. Mice were anesthetized with isoflurane and blood glucose was sampled via the tail vein (Accu-Chek Aviva; Roche Diagnostics, Mannheim, Germany). [^{18}F]-FDG activity of approximately 15-20 MBq was administered via the penile vein. PET emission data were acquired for 30 min followed by a 15 min transmission scan to correct for photon attenuation and scatter. PET images were reconstructed and analyzed using Inveon Research Workplace (Siemens, USA). Three consecutive regions of interest were selected from the left ventricular myocardium in the frontal and coronal planes. The myocardial [^{18}F]-FDG uptake was calculated as a standardized uptake value (SUV) and corrected for body weight and time of decay before and after tracer injection; SUV = activity measured / [activity injected/body weight (g)]. For analysis of the HFD study, the window level was set at an SUV of 20 for all animals to remove bias. The SUV was adjusted to a setting of 8 for all mice in the STZ study in order to account for the broader range of detected [^{18}F]-FDG levels.

RNA isolation and quantitative real-time PCR

Total RNA extracted from tissue using TRIzol reagent (Invitrogen, Carlsbad, CA, USA) was reverse transcribed to cDNA (RNeasy Mini kit; Qiagen Inc, Valencia, CA, USA) and subjected to quantitative real-time polymerase chain reaction (PCR) (C1000 Thermal Cycler CFX384; Bio-Rad Laboratories, Veenendaal, The Netherlands). Transcript levels were quantified and normalized to the invariant transcript, *36b4*. Primer sequences used for quantitative PCR analyses are listed in ESM Table 2.

Immunoblotting

Cardiac protein lysates were prepared as previously described [3]. Western blots analyses were performed using primary and secondary antibodies using the following: anti-human LXRα (Perseus Proteomics); AMPKα, phospho-AMPKα (Thr172), AKT, phospho-AKT (Ser473), phospho-AS160 (Thr642), GLUT4, p44/42 MAPK (ERK1/2), S6 kinase, phospho-S6 (Ser235/236) (Cell Signaling); LKB1(Abcam); CaMKKβ (ThermoScientific); phospho-ERK (Santa Cruz), anti-phospho-p70S6 Kinase (T389) (R&D Systems); glyceraldehyde-3-phosphate dehydrogenase (Fitzgerald, USA); anti-α-Tubulin (Sigma Aldrich); rabbit anti-mouse immunoglobulins/HRP, goat anti-rabbit immunoglobulins/HRP (Dako, Denmark). Signals were detected by ECL (PerkinElmer, Waltham, MA, USA), and densitometry was quantified with ImageQuant LAS 4000 (GE Healthcare Europe, Diegem, Belgium).

Biochemical assays

Plasma analyses were performed from collection of blood following 4 hours of fasting to assess plasma triacylglycerol (Roche Diagnostics) and insulin using an enzyme-linked immunosorbent assay (ELISA) kit (cat. nr. 80-INSMSU-E01-; ALPCO Diagnostics, New Hampshire, USA). Myocardial lipids were extracted from frozen LV tissue according to Bligh & Dyer protocol [4], and triacylglycerol was measured with a commercial kit (Roche Diagnostics). Cardiac glycogen content was determined using EnzyChrom Glycogen Assay kit (BioAssay Systems, Hayward, CA, USA), as previously described [1].

Histological analysis

Mid-ventricular cross-sectional slices were immersed in Tissue-Tek for cryopreservation (Sakura Finetek, USA). For neutral lipid detection, 4 µm frozen mid-ventricular sections were stained with Oil red O (Sigma Aldrich) and imaged at 40X magnification (ScanScope, Aperio Technologies, Vista, CA, USA).

Chromatin immunoprecipitation (ChIP) assay

Identification of putative DR4 LXREs in the promoter region of *Anp* (also known as *Nppa*) and *Bnp* (also known as *Nppb*) genes was performed using bioinformatics tools. In both mouse and rat, several potential LXREs were identified within 50 kb upstream and downstream of the transcriptional start site (TSS).

Chromatin immunoprecipitation (ChIP) experiments were performed in both isolated neonatal rat ventricular myocytes (NRVMs) and in hearts from *Lxrα*-Tg and WT mice using the Pierce Agarose ChIP Kit (Thermo Scientific, Rockford, IL, USA). NRVMs were isolated and cultured from 1-3 day old Sprague-Dawley pups, as previously described [3, 5]. NRVMs were transfected with adenoviral constructs containing either murine LXRα (Ad-LXRα), LXRα-specific siRNA (si-LXRα), or GL2 control viruses (Ad-cont), as previously described [5]. Oligonucleotide sequences used for cloning are presented in ESM Table 3. For preparation of chromatin in NRVMs, cells were fixed with paraformaldehyde (PFA; 1% final concentration) for 10 min at room temperature to cross-link proteins to DNA. Cross-linking was terminated by addition of 125 mM glycine for 5 min at room temperature. Following incubation, cells were washed twice with ice-cold phosphate-buffered saline (PBS), detached, and the cell suspension pelleted by centrifugation at 3000 *g* for 5 min.

For preparation of chromatin from tissue samples, murine hearts were flushed with ice-cold PBS and harvested. Two hearts were pooled per genotype; the experiment was conducted for two murine preparations per genotype. Tissue samples were finely minced in 5 ml of Dulbecco’s Modified Eagle’s Medium (DMEM; 4.5 g/l glucose) on ice. Cross-linking was performed by addition of 1.5% PFA and incubation for 20 min at room temperature on a shaker. The reaction was stopped with 125 mM glycine for 5 min at room temperature, followed by centrifugation of the samples at 4500 *g* for 5 min and two sequential washes with ice-cold PBS containing phenylmethylsulfonyl fluoride (PMSF). The pelleted tissue was then subjected to disaggregation using a T 25 digital Ultra Thurrax disperser (IKA, Staufen, Germany) in PBS supplemented with protease inhibitors and PMSF, then centrifuged at 4500 *g* for 5 min

at 4°C. The supernatant was removed and 10 ml of a cell lysis buffer (10 mM Tris-HCl, pH 8.0, 10 mM NaCl, 0.2% NP40) was added to the pellet, vortexed, and centrifuged 4500 *g* for 5 min at 4°C.

Cell pellets obtained from either NRVMs or tissue preparation were then resuspended in lysis buffer containing protease inhibitors, and incubated for 10 min on ice. Samples were centrifuged at 9000 *g* for 3 min at 4°C, followed by MNase digestion with Micrococcal Nuclease for 15 min at 37°C. Digestion was stopped by incubating samples in an MNase Stop Solution on ice for 5 min. Nuclei were recovered by centrifugation at 9000 *g* for 5 min and resuspension of the pellet in lysis buffer. Samples were centrifuged again, and the supernatant containing digested chromatin was used for subsequent immunoprecipitation (IP) using antibodies for LXRα.

From each chromatin sample, 10% was removed for input control and the remaining sample was split for anti-LXRα IP (1 μg/μl anti-human LXRα, Perseus Proteomics), or nonspecific rabbit IgG (provided by manufacturer) as a negative control IP. The remaining procedure was performed according to manufacturer's instructions, using supplied buffers (Thermo Scientific). Briefly, IPs were incubated overnight on a rocking platform at 4°C in IP dilution buffer. The antibody-protein-DNA complex was pulled down with ChIP Grade Protein A/G Plus agarose beads, and washed sequentially. DNA-protein complexes were eluted, and proteins were digested with Proteinase K at 65°C for 1.5 hrs. Protein-bound immunoprecipitated DNA was reverse cross-linked, and immunoprecipitated DNA fragments were eluted and purified for quantitative real-time PCR detection.

From 45-50 µl extraction volume, 3 µl was used as a template for PCR amplification (35 cycles), and real-time PCR was performed with SYBR green on C1000 Thermal Cycler CFX384 (Bio-Rad Laboratories, Veenendaal, The Netherlands). The sets of primers used to amplify the regions of interest are shown in ESM Table 4. Agarose gel-based electrophoresis was performed for visualization and

quantification of ChIP results (Image J, NIH, Bethesda, MD, USA). Input samples were normalized to *36b4* levels.

Statistics

Data are expressed as means ± SEM. For group comparisons, one-way ANOVA was performed followed by Tukey's post hoc analysis. When the data were not normally distributed according to Shapiro Wilk test for normality, Kruskal Wallis test, followed by a Mann Whitney *U* test for individual comparison of means, were performed. A value cut-off of $p<0.05$ was considered statistically significant. Statistical analyses were performed using IBM SPSS Statistics 22 (Chicago, IL, USA).

References

[1] Cannon MV, Sillje HHW, Sijbesma JWA, et al. (2015) Cardiac LXRα protects against pathological cardiac hypertrophy and dysfunction by enhancing glucose uptake and utilization. EMBO Mol Med 7:1229-1243

[2] Yu L, Ruifrok WP, Meissner M, et al. (2013) Genetic and pharmacological inhibition of galectin-3 prevents cardiac remodeling by interfering with myocardial fibrogenesis. Circ Heart Fail 6:107-117

[3] Cannon MV, Yu H, Candido WM, et al. (2015) The liver X receptor agonist AZ876 protects against pathological cardiac hypertrophy and fibrosis without lipogenic side effects. Eur J Heart Fail 17:273-282

[4] BLIGH EG, DYER WJ (1959) A rapid method of total lipid extraction and purification. Can J Biochem Physiol 37:911-917

[5] Lu B, Mahmud H, Maass AH, et al. (2010) The Plk1 inhibitor BI 2536 temporarily arrests primary cardiac fibroblasts in mitosis and generates aneuploidy in vitro. PLoS One 5:e12963

ESM Table 2. Murine gene primers for real-time PCR

Gene	Forward primer (5'-3')	Reverse primer (5'-3')
Myh6	GTTAACCAGAGTTTGAGTGACA	CCTTCTCTGACTTTCGGAGGTACT
Myh7	ATGTGCCGGACCTTGGAAG	CCTCGGGTTAGCTGAGAGATCA
Acta1	TGCCATGTATGTGGCTATCCA	TCCCCAGAATCCAACACGAT
Rcan1	GCTTGACTGAGAGAGCGAGTC	CCACACAAGCAATCAGGGAGC
Cd36	CTGTGTTTGGAGGCATTC	AGCAGTGGTTCCTTCTTC
Acc2	ATCTGAAGCGGGACTCTG	AGCTGAGCCACCTGTATC
Cpt1a	CCTGCATTCCTTCCCATTTG	AGTCATGGAAGCCTCATACG
Cpt1b	CCCATGTGCTCCTACCAG	CACGTGCCTGCTCTCTGA
Glut1	GGTGTGCAGCAGCCTGTGTA	GACGAACAGCGACACCACAGT
Hk2	GGACGGGACACTGTACAAG	GCCACAGCAGTGATGAGAG
Nppa	ATGGGCTCCTTCTCCATCAC	TCTACCGGCATCTTCTCCTC
Nppb	AAGTCCTAGCCAGTCTCCAGA	GAGCTGTCTCTGGGCCATTTC
36b4	AAGCGCGTCCTGGCATTGTC	GCAGCCGCAAATGCAGATGG

ESM Table 3. Primer sequences used for cloning

Gene	5'-3'
LXRα forward	GTTGGATCCACCATGTCCTTGTGGCTGGAGG
LXRα reverse	GAACTCGAGTCATTCGTGGACATCCCAGATC
si-LXRα forward	GATCCCGGAGTGTCGCCTTCGCAAATTCAAGAGATTTGCGAAGGCGACACT CCTTTTTGGAAA
si-LXRα reverse	AGCTTTTCCAAAAAGGAGTGTCGCCTTCGCAAATCTCTTGAATTTGCGAAGG CGACACTCCGG

ESM Table 4. Primer sequences for LXR response elements in chromatin immunoprecipitation analysis

LXRE	Forward primer (5'-3')	Reverse primer (5'-3')
Mouse:		
LXRE 6	GGACTGAGGGGTCACTCAT	CCCTTCCGTCCTGTCACATAG
LXRE 14	TAGACTTCCTCCTGCCATCC	TGCCCTGCAATGACACTG
Rat:		
LXRE 14	CTTCCTCCTGCCATCTAGTG	CTACAGAAACAGCAAGCCTG
