# Supplementary material for: LXRα improves myocardial glucose tolerance and reduces cardiac hypertrophy in a mouse model of obesity-induced type 2 diabetes
Source: Diabetologia. 2015 Dec 18;59:634–43. doi: 10.1007/s00125-015-3827-x (PMC4742491; doi:10.1007/s00125-015-3827-x)
Supplement: Supplementary file 2 — (PDF 367 kb) [file 125_2015_3827_MOESM2_ESM.pdf]

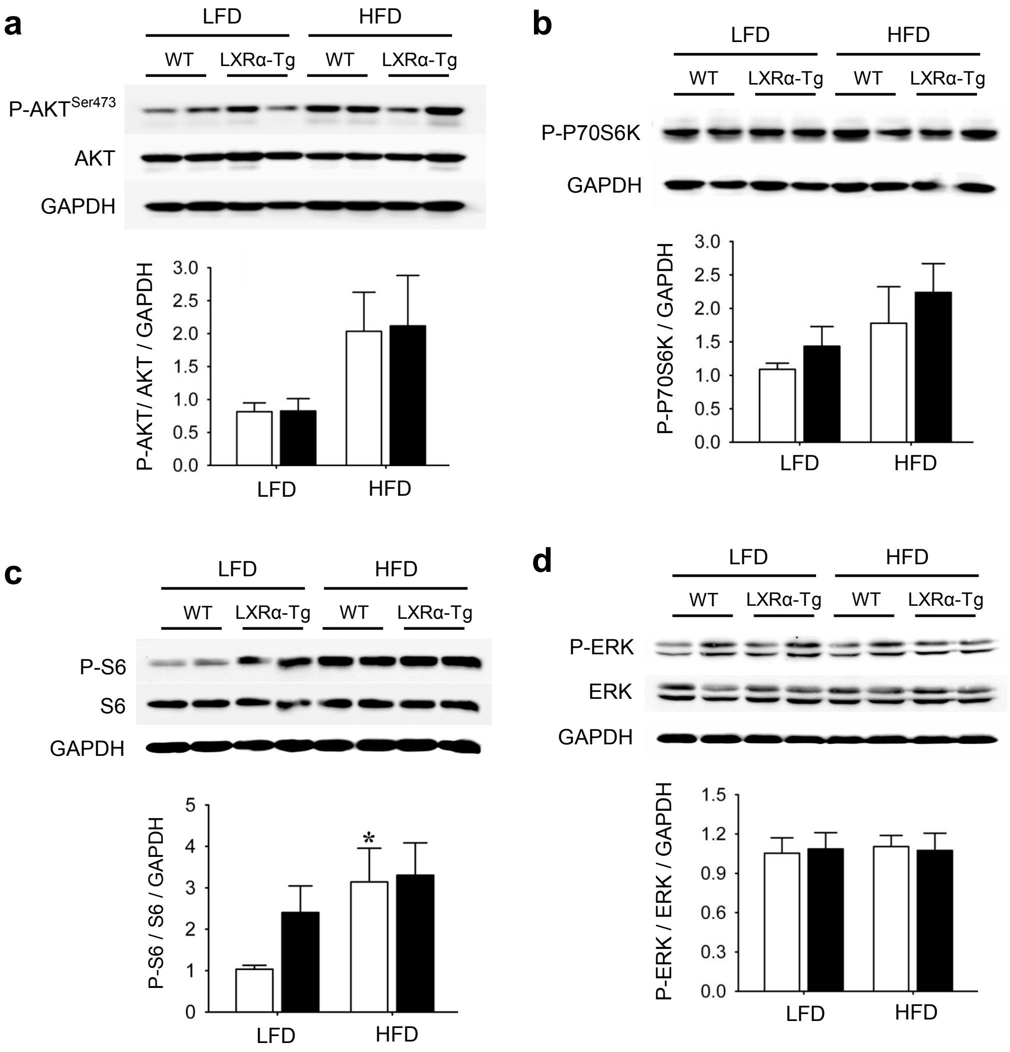

ESM Fig. 1

Assessment of hypertrophic signaling pathways in mice subjected to HFD. (**a-d**) WT (white bars), *Lxrα*-Tg (black bars). Western blot analysis was performed to measure protein levels in LV tissue for the following phosphorylated proteins, (**a**) AKT ^{Ser473}, (**b**) P70S6 kinase, (**c**) ribosomal protein S6 kinase, and (**d**) p44/42 MAPK/ERK, normalized to GAPDH and expressed as fold change; n=7-8/group. Data are means ± SEM**;** *p<0.05 versus respective control.
