# Supplementary material for: LXRα improves myocardial glucose tolerance and reduces cardiac hypertrophy in a mouse model of obesity-induced type 2 diabetes
Source: Diabetologia. 2015 Dec 18;59:634–43. doi: 10.1007/s00125-015-3827-x (PMC4742491; doi:10.1007/s00125-015-3827-x)
Supplement: Supplementary file 3 — (PDF 349 kb) [file 125_2015_3827_MOESM3_ESM.pdf]

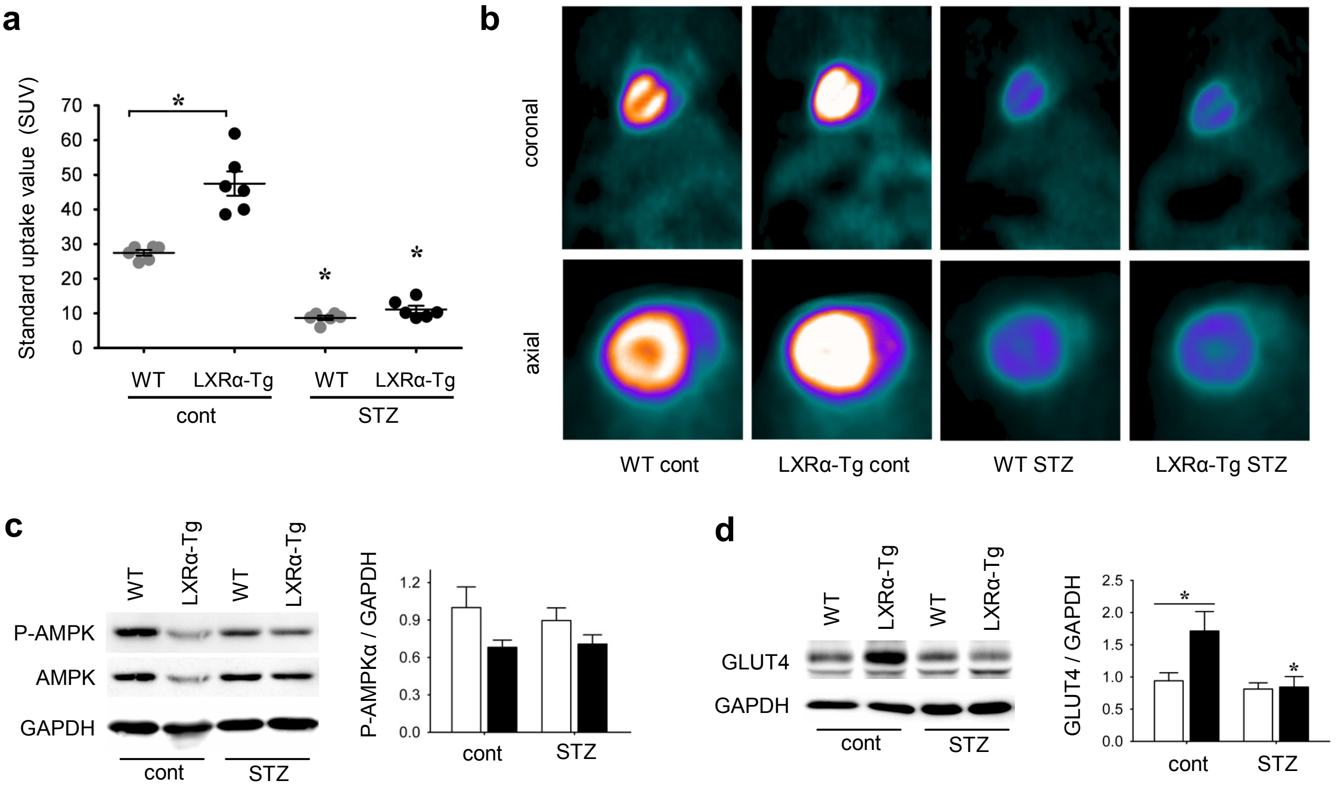

ESM Fig. 2

Cardiac glucose uptake levels are impaired in streptozotocin (STZ)-treated mice. (**a**) Standard uptake values (SUV) were measured using ^{18}F-FDG and microPET imaging in WT and *Lxrα*-Tg mice fed normal chow for 12 weeks; n=6/group. Data are means ± SEM; *p<0.01 versus respective control, *p<0.01 versus WT. (**b**) Representative PET images in coronal and axial planes. For the analysis, window level settings were adjusted in order to accommodate the broader range of FDG levels. (**c**-**d**) WT (white bars), *Lxrα*-Tg (black bars). Quantification of (**c**) AMPKα phosphorylation and (**d**) GLUT4 protein levels in LV tissue normalized to GAPDH; n=6/group. Data are means ± SEM; *p<0.05 versus respective control, *p<0.05 versus WT.
