# Supplementary material for: LXRα improves myocardial glucose tolerance and reduces cardiac hypertrophy in a mouse model of obesity-induced type 2 diabetes
Source: Diabetologia. 2015 Dec 18;59:634–43. doi: 10.1007/s00125-015-3827-x (PMC4742491; doi:10.1007/s00125-015-3827-x)
Supplement: Supplementary file 4 — (PDF 378 kb) [file 125_2015_3827_MOESM4_ESM.pdf]

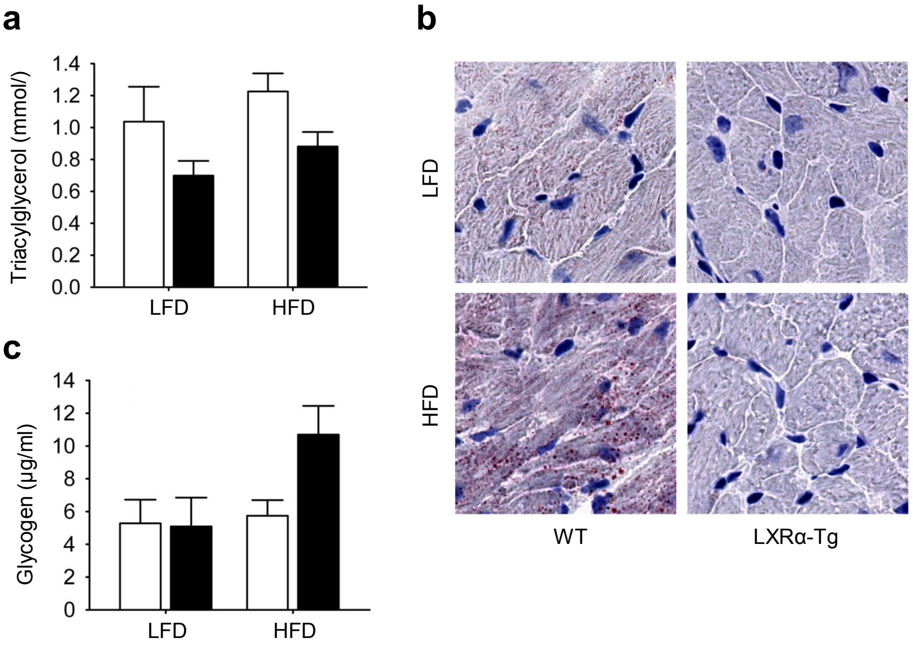

ESM Fig. 3

Determination of cardiac triacylglycerol and glycogen content in response to HFD intervention.

(**a**, **c**) WT (white bars), *Lxrα*-Tg (black bars). (**a**) Cardiac triacylglycerol; n=5-7/group. (**b**) Representative LV histological sections stained with Oil red O for the detection of neutral lipids. (**c**) Cardiac glycogen content; n=6/group**.** Data are means ± SEM; *p*=NS**.**
