# Supplementary material for: LXRα improves myocardial glucose tolerance and reduces cardiac hypertrophy in a mouse model of obesity-induced type 2 diabetes
Source: Diabetologia. 2015 Dec 18;59:634–43. doi: 10.1007/s00125-015-3827-x (PMC4742491; doi:10.1007/s00125-015-3827-x)
Supplement: Supplementary file 5 — (PDF 225 kb) [file 125_2015_3827_MOESM5_ESM.pdf]

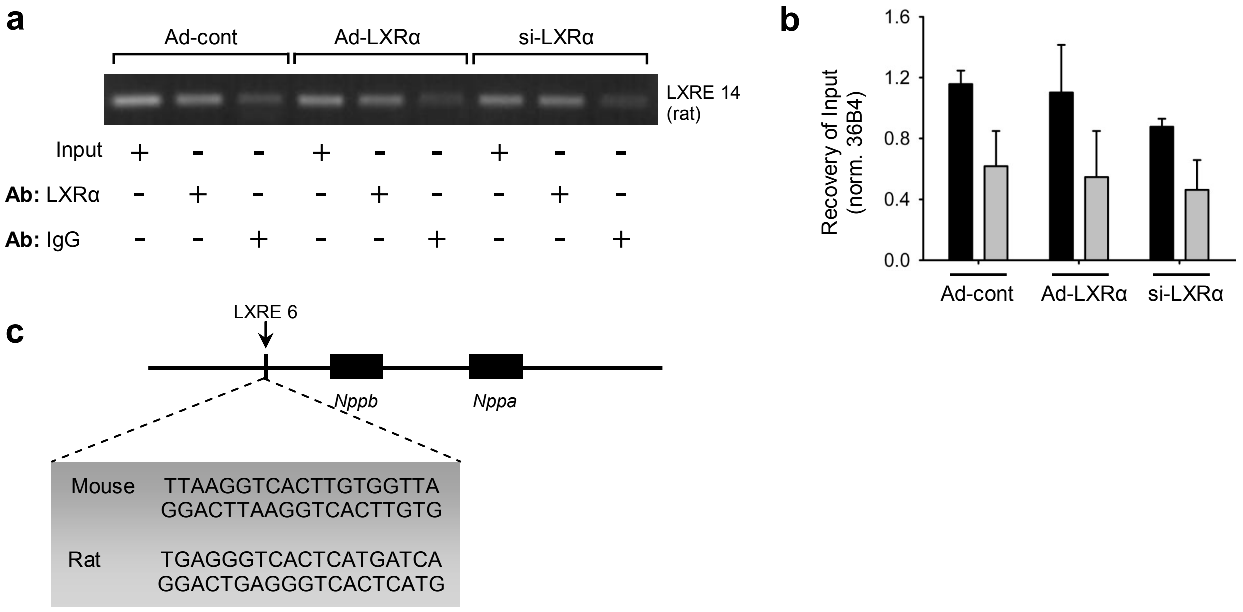


**ESM Fig. 4**

LXRα is recruited to the ANP/BNP region in rat cardiomyocytes. (**a**) Chromatin immunoprecipitation assays were performed using isolated rat cardiomyocytes to analyze the ANP/BNP region. Rat cardiomyocytes were transfected with Ad-LXRα or si-LXRα to cause LXRα overexpression and deficiency, respectively. The results are for LXRE 14 and are representative of two independent cardiomyocyte isolations. (**b**) Quantification of the RT-PCR fragments were analyzed from gel electrophoresis. Ab: LXRα (black bars), Ab: IgG (gray bars). Non-specific IgG antibodies served as negative control. (**c**) Location of LXRE 6 is ~22 kb upstream of the ANP (*Nppa*) transcription start site, and ~6.5 kb upstream of BNP (*Nppb*) promoter. LXRE 6 binding site sequences were identified using the JASPAR database, and analysis revealed a highly conserved double binding site sequence for both the rat and mouse. Ab, antibody; mu, murine.
