# Supplementary material for: LXRα improves myocardial glucose tolerance and reduces cardiac hypertrophy in a mouse model of obesity-induced type 2 diabetes
Source: Diabetologia. 2015 Dec 18;59:634–43. doi: 10.1007/s00125-015-3827-x (PMC4742491; doi:10.1007/s00125-015-3827-x)
Supplement: Supplementary file 6 — (PDF 455 kb) [file 125_2015_3827_MOESM6_ESM.pdf]

ESM Table 1. Biometric, echocardiographic, and hemodynamic variables of *Lxrα*-Tg and WT mice subjected to 12 weeks streptozotocin-induced type 1 diabetes

Variable	Control		STZ	
	**WT**	***Lxrα*-Tg**	**WT**	***Lxrα* –Tg**
Post mortem organ weight	(n=10)	(n=10)	(n=11)	(n=10)
Body weight (g)	30.6 ± 0.5	32.4 ± 0.9	25.4 ± 0.6 ***	25.1 ± 0.3 ***
LV/tibia (mg/mm)	7.0 ± 0.2	6.9 ± 0.2	6.2 ± 0.2 **	5.6 ± 0.1 ***
Kidney/tibia (mg/mm)	25.4 ± 0.6	27.3 ± 0.8	31.1 ± 0.7 ***	30.1 ± 0.8 *
Liver/tibia (mg/mm)	93.9 ± 5.7	93.1 ± 3.4	105.2 ± 3.0 *	104.5 ± 2.0 *
Echocardiography	(n=10)	(n=10)	(n=11)	(n=10)
Heart rate (bpm)	431 ± 14	422 ± 7	374 ± 9 *	340 ± 19 **
LVCO/BW (ml $min^{-1}g^{-1}$)	1.00 ± 0.05	0.98 ± 0.07	0.65 ± 0.06 ***	0.94 ± 0.09 ***
Stroke volume (µl)	69.1 ± 3.5	73.2 ± 4.4	47.7 ± 2.1 ***	63.5 ± 4.1 ††
Interventricular septum (mm)				
Diastole	0.73 ± 0.02	0.68 ± 0.01	0.66 ± 0.01 *	0.67 ± 0.02
Systole	1.55 ± 0.07	1.57 ± 0.04	1.29 ± 0.04 **	1.40 ± 0.06
LV posterior wall (mm)				
Diastole	0.74 ± 0.02	0.69 ± 0.02	0.72 ± 0.02	0.78 ± 0.06
Systole	1.53 ± 0.06	1.33 ± 0.07	1.09 ± 0.08 **	1.23 ± 0.08
LV internal diameter (mm)				
Diastole	4.00 ± 0.03	3.87 ± 0.06	3.69 ± 0.07 **	3.73 ± 0.10
Systole	2.33 ± 0.02	2.27 ± 0.05	2.50 ± 0.07	2.35 ± 0.10
Fractional shortening (%)	41.9 ± 0.5	41.4 ± 0.6	32.6 ± 1.0 ***	37.3 ± 1.5 ***
E velocity (m/s)	0.79 ± 0.04	0.72 ± 0.02	0.59 ± 0.03 **	0.50 ± 0.03
A velocity (m/s)	0.51 ± 0.04	0.41 ± 0.02	0.39 ± 0.01 *	0.34 ± 0.02 *
E/A ratio	1.6 ± 0.1	1.8 ± 0.1	1.5 ± 0.1	1.5 ± 0.1 *
Deceleration time (ms)	47.2 ± 2.4	45.3 ± 1.8	60.5 ± 2.5 **	51.9 ± 3.7 **
Hemodynamics	(n=9)	(n=9)	(n=11)	(n=9)
Aortic systolic pressure (mmHg)	89.4 ± 2.7	85.4 ± 3.0	92.5 ± 3.2	86.8 ± 1.7
Aortic diastolic pressure (mmHg)	55.5 ± 2.4	56.7 ± 2.2	65.3 ± 2.8 *	62.0 ± 1.8
Mean arterial pressure (mmHg)	66.8 ± 2.3	66.3 ± 2.5	74.3 ± 2.9	70.3 ± 1.8
LV end-systolic pressure (mmHg)	98.6 ± 3.7	91.2 ± 2.8	103.2 ± 3.5	90.2 ± 3.6
LV end-diastolic pressure (mmHg)	11.5 ± 3.2	13.2 ± 3.0	17.7 ± 2.1	16.0 ± 2.1
dP/dt_{max} (mmHg)	8661 ± 396	7531 ± 398	6133 ± 249 ***	5667 ± 263 **
dP/dt_{min} (mmHg)	-8150 ± 484	-6503 ± 376	-5785 ± 374 **	-5336 ± 477
Blood chemistry	(n=10)	(n=10)	(n=11)	(n=10)
Blood glucose (mmol/l)	13.8 ± 1.1	15.2 ± 0.9	33.4 ± 0.5 ***	33.7 ± 0.3 ***
Plasma insulin (pmol/l)	0.24 ± 0.07	0.26 ± 0.05	0.13 ± 0.02	0.15 ± 0.02

Data are expressed as means ± SEM. *$p<0.05$, **$p<0.01$, ***$p<0.001$, STZ versus corresponding control group; †† $p<0.01$, WT vs *Lxrα*-Tg mice.
STZ, streptozotocin; LV, left ventricular; BW, body weight; bpm, beats per minute.
